# Levels of evidence and grades of recommendation supporting European society for medical oncology clinical practice guidelines

**DOI:** 10.32604/or.2024.048948

**Published:** 2024-04-23

**Authors:** MARKO SKELIN, BRUNA PERKOV-STIPIČIN, SANJA VUŠKOVIĆ, MARINA ŠANDRK PLEHAČEK, ANE BAŠIĆ, DAVID ŠARČEVIĆ, MAJA ILIĆ, IVAN KREČAK

**Affiliations:** 1 Pharmacy Department, General Hospital of Šibenik-Knin County, Šibenik, Croatia; 2 Faculty of Medicine, University of Rijeka, Rijeka, Croatia; 3 Undergraduate Study in Nursing, University of Applied Sciences, Šibenik, Croatia; 4 Department of Oncology, University Hospital Center Zagreb, Zagreb, Croatia; 5 Pharmacies Joukhadar, Sveta Nedelja, Croatia; 6 Pharmacies Prima Pharme, Zagreb, Croatia; 7 Pharmacy Department, General Hospital Zadar, Zadar, Croatia; 8 Pharmacy Department, General Hospital Pula, Pula, Croatia; 9 Department of Internal Medicine, General Hospital of Šibenik-Knin County, Šibenik, Croatia

**Keywords:** ESMO guidelines, Clinical practice guidelines, Level of evidence, Grade of recommendation

## Abstract

**Background:**

The European Society for Medical Oncology (ESMO) guidelines are among the most comprehensive and widely used clinical practice guidelines (CPGs) globally. However, the level of scientific evidence supporting ESMO CPG recommendations has not been systematically investigated. This study assessed ESMO CPG levels of evidence (LOE) and grades of recommendations (GOR), as well as their trends over time across various cancer settings.

**Methods:**

We manually extracted every recommendation with the Infectious Diseases Society of America (IDSA) classification from each CPG. We examined the distribution of LOE and GOR in all available ESMO CPG guidelines across different topics and cancer types.

**Results:**

Among the 1,823 recommendations in the current CPG, 30% were classified as LOE I, and 43% were classified as GOR A. Overall, there was a slight decrease in LOE I (−2%) and an increase in the proportion of GOR A (+1%) in the current CPG compared to previous versions. The proportion of GOR A recommendations based on higher levels of evidence such as randomized trials (LOE I–II) shows a decrease (71% *vs*. 63%, *p* = 0.009) while recommendations based on lower levels of evidence (LOE III–V) show an increase (29% *vs*. 37%, *p* = 0.01) between previous and current version. In the current versions, the highest proportion of LOE I (42%) was found in recommendations related to pharmacotherapy, while the highest proportion of GOR A recommendations was found in the areas of pathology (50%) and diagnostic (50%) recommendations. Significant variability in LOE I and GOR A recommendations and their changes over time was observed across different cancer types.

**Conclusion:**

One-third of the current ESMO CPG recommendations are supported by the highest level of evidence. More well-designed randomized clinical trials are needed to increase the proportion of LOE I and GOR A recommendations, ultimately leading to improved outcomes for cancer patients.

## Introduction

The recent proliferation of research publications has caused clinical practice guidelines (CPG) to emerge as the most important tool used by healthcare providers for making evidence-based clinical decisions. CPG combines scientific evidence and clinical judgment to develop recommendations that help practitioners with decisions about appropriate care for specific patients’ circumstances [1–4]. Guidelines recommendations are guided by clinical scenarios and are often assumed to be the epitome of evidence-based medicine. One should bear in mind, that guideline recommendations may also be influenced by personal or organizational preferences regarding the risks and benefits of different medical interventions [5]. The quality of evidence that supports each recommendation varies from high-quality (evidence from at least one large randomized controlled trial of good methodological quality and therefore with a low potential for bias, or meta-analyses of well-conducted randomised controlled trials without heterogeneity) to low-quality (studies without control group, case reports, expert opinions).

Oncology is a fast-growing field characterized by rapid drug development and emerging therapeutic possibilities. Consequently, the question arises whether research and the quality of guidelines can keep up with such rapid growth. European Society for Medical Oncology (ESMO) guidelines are one of the most comprehensive and widely used clinical practice guidelines in the world. For two decades ESMO has released CPGs that have integrated evidence-based medicine framework by assigning a level of evidence (LOE) to each recommendation, together with the grade of recommendation (GOR) supporting a particular recommendation. Recently, ESMO guidelines started to use a grading schema based on the level of evidence and grade of recommendation according to qualification systems developed by the Infectious Diseases Society of America (IDSA) [6]. IDSA represents a panel of experts who perform a systematic review of the available evidence and use the GRADE process to develop evidence-based recommendations to help practitioners and patients make decisions about appropriate health care for various medical settings [7]. This feature enables comparison of different guideline topics together with an investigation of changes in guideline recommendations over time. An increase in the quantity of scientific research concerning malignant disease published in recent years should have resulted in more certainty in guideline recommendations and increased levels of evidence. Yet, recent research has revealed a lack of high-quality research present in the CPG of the National Comprehensive Cancer Network (NCCN). This particular network is a set of guidelines developed and updated by 61 individual panels from the 33 NCCN member institutions which is considered a standard for clinical direction and policy in USA cancer care [8,9]. The same issue was also found in other medical settings [10,11]. However, it is unknown whether ESMO CPG may have also been affected by this trend.

The aim of this analysis was to perform an analysis of ESMO clinical practice guidelines with the intent to assess levels of evidence, grades of recommendations, and their changes over time across different cancer settings.

## Methods and Materials

In our analysis we have included 41 topic (Malignant Pleural Mesothelioma, Endometrial Cancer, Bladder Cancer, Brain Metastasis from Solid Tumours, Leptomeningeal Metastasis, Penile Cancer, Thymic Epithelial Tumors, Acute Lymphoblastic Leukaemia, Extranodal Diffuse large B-cell lymphoma (DLBCL) and Primary Mediastinal B-Cell Lymphoma, Hairy Cell Leukaemia, Peripheral T-cell Lymphomas, Philadelphia Chromosome Negative Chronic MPNs, Gestational Trophoblastic Disease, Hereditary Gastrointestinal Cancer, Marginal Zone Lymphomas, Prostate Cancer, Renal Cell Carcinoma, Gastric Cancer, Rectal Cancer, Soft Tissue and Visceral Sarcomas, Oesophageal Cancer, Nasopharyngeal Carcinoma, Small-Cell Lung Cancer, Non-Small-Cell Lung Cancer, Metastatic Small-Cell Lung Cancer, Melanoma, Metastatic Colorectal Cancer, Localised Colon Cancer, Hepatocellular Carcinoma, Gastrointestinal Stromal Tumor, Metastatic Breast Cancer, Early Breast Cancer, Bone Sarcomas, Waldenstrom Macroglobulinaemia, Myelodysplastic Syndromes, Multiple Myeloma, Mantle Cell Lymphoma, Chronic Lymphocytic Leukaemia, Hodgkin Lymphoma, Follicular Lymphoma, Acute Myeloid Leukaemia). Three available versions (first, previous and current) of ESMO guidelines from each topic issued from May 2005 to October 2022 were downloaded and abstracted by DŠ, MŠ, AB, BPS, and MI and validated by MS, SV and IK. The analysis included the percentage of recommendations within each class of recommendation and the distribution of the level of evidence designations across guidelines. Definitions of IDSA classifications are shown in Tables 1 and 2.

**Table 1:** Level of evidence classification by IDSA

Level of evidence	
I	Evidence from at least one large randomised, controlled trial of good methodological quality (low potential for bias) or meta-analyses of well-conducted randomised trials without heterogeneity
II	Small randomised trials or large randomised trials with suspicion of bias (lower methodological quality) or meta-analyses of such trials or of trials with demonstrated heterogeneity
III	Prospective cohort studies
IV	Retrospective cohort studies or case–control studies
V	Studies without control group, case reports, expert opinions

**Table 2:** Grade of recommendation classification by IDSA

Grades of recommendation	
A	Strong evidence for efficacy with a substantial clinical benefit, strongly recommended
B	Strong or moderate evidence for efficacy but with limited clinical benefit, is generally recommended
C	Insufficient evidence for efficacy or benefit does not outweigh the risk or the disadvantages (adverse events, costs, etc.), optional
D	Moderate evidence against efficacy or for adverse outcome, generally not recommended
E	Strong evidence against efficacy or for adverse outcome, never recommended

The data regarding pharmacotherapy, radiation, supportive therapy, surgery, tumor pathology and genetic alterations, screening methods, diagnostic procedures, staging procedures, follow-up, disease stages, cancer types (hematological *vs*. solid tumors) and transplantation was extracted from analysed guidelines. Rare tumors were classified according to the Orphanet database [12]. Guidelines marked as “genetic alterations” contain information regarding specific genetic molecules or markers that should be tested in order to establish a diagnosis or to decide about further treatment; those marked as “pathology” contain information about the pathohistological classification of disease. Finally, the authors focused specifically on the recommendations regarding individual cancer types.

The authors searched each guideline to find recommendations stated in the summary of recommendations table. If no summary table was available, the authors included recommendations that were clearly displayed statements highlighted in each guideline document and were separated from the remainder of the text. In case they were not clearly separated from the rest of the text, the authors searched throughout the text and extracted recommendations with their affiliated level of evidence and grade of recommendation. In case there were two levels of evidence or grades of recommendations affiliated with one recommendation, the most favourable one was included in the analysis. Additional screening of the guidelines was performed in order to make sure all relevant data was included in the analysis. Abstraction of the data included in this analysis was not subject to any judgment by the abstractors.

**Figure 1:**
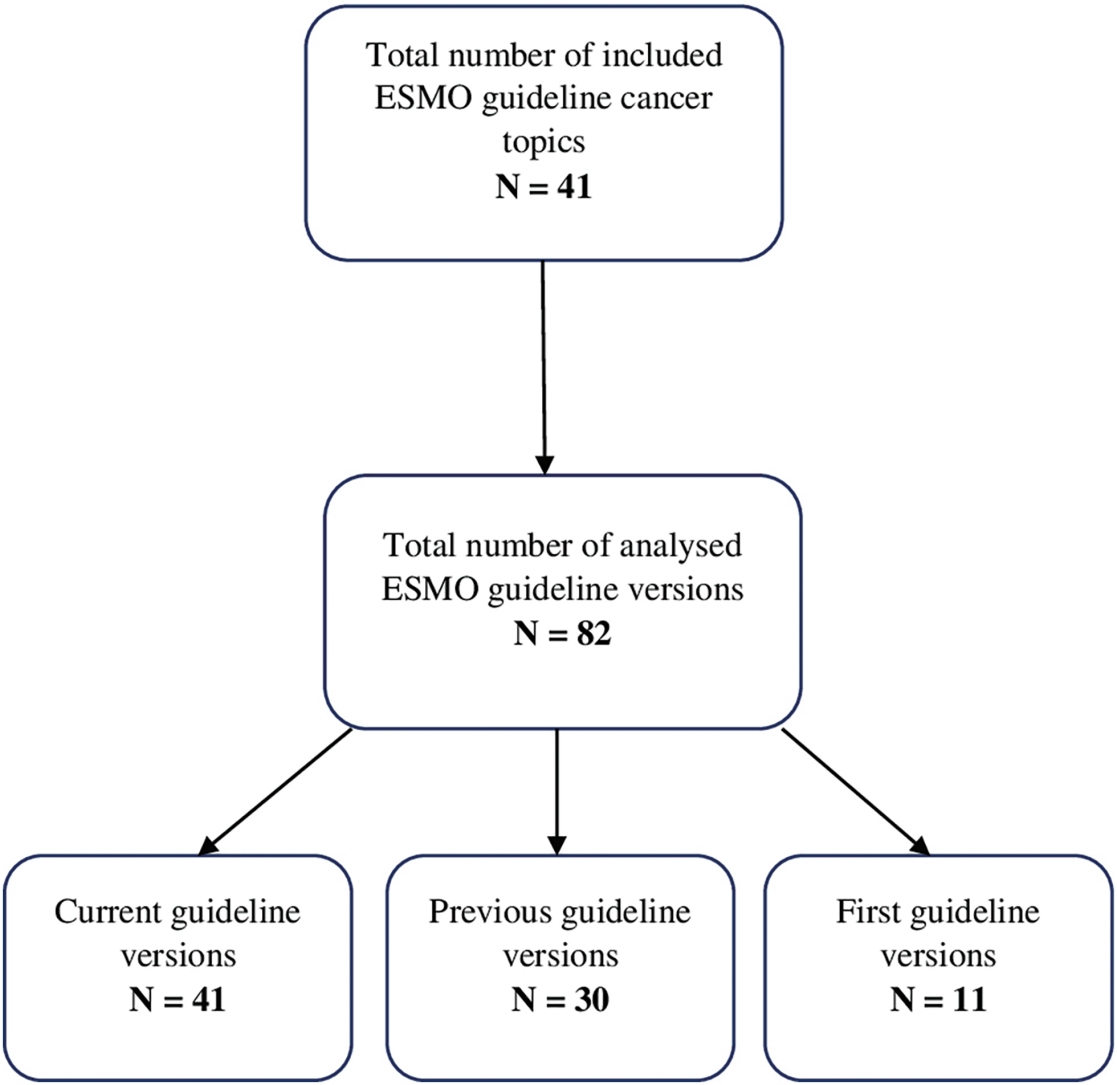
Flowchart of included guideline versions.

Three versions of guidelines (first, previous and current) were chosen so we can analyse their changes over the shortest and longest period of time. Current guidelines were defined as those posted on the ESMO website (www.esmo.org/guidelines) on December 10^th^, 2022. Previous versions of these guidelines were marked as those referenced in the current guidelines. Additionally, we have searched the ESMO website and PubMed database in order to find the first and previous editions of analysed guidelines. The analysis included only comprehensive guideline documents including focused updates. We excluded guidelines without IDSA classification. The flowchart diagram of the included studies is presented in Fig. 1.

The main aims of this analysis were; 1) to report how many recommendations in ESMO guidelines are supported with the highest quality evidence (Level I) and grade of recommendation (A), 2) to assess the distribution of levels of evidence and grades of recommendation, and 3) to analyse trends in the levels of evidences and grades of recommendations over time among the three guidelines (first, previous, and the current) across different cancer fields.

The statistical test used for comparing the proportions between previous and current versions of guidelines was the two proportions z-test. *p*-values less than 0.05 were considered to be statistically significant. The data was analysed and graphs were constructed using R (version 2022.12.0+353).

## Results

The analysis included 82 guidelines on 41 topics that were published from 2005–2022. There were 41 current, 30 previous and 11 first versions of the ESMO CPG. From these 82 guidelines, we have extracted and categorized a total of 3068 recommendations. As shown in Suppl. Fig. S1, the total number of recommendations has significantly risen from the first, previous and current guidelines, with 360, 885, and 1823 recommendations, respectively. Therefore, we have noticed an increase of 106% in the number of recommendations from the previous to the current version of guidelines. All recommendations about their respective levels of evidence and grades of recommendation are shown in Table 3 and Suppl. Tables S1 and S2.

**Table 3:** ESMO clinical practice guidelines and associated level of evidence I and grade of recommendation A

LOE I GOR A	First LOE I	Previous LOE I	Current LOE I	Difference (current *vs*. previous) LOE I	GOR A first	GOR A previous	GOR A current	Difference (current *vs*. previous) GOR A
All	133 (37%)	284 (32%)	550 (30%)	−2%	139 (39%)	375 (42%)	791 (43%)	1%
Acute Myeloid Leukaemia	X	11 (48%)	8 (16%)	−32%	X	10 (44%)	17 (33%)	−11%
Follicular Lymphoma	X	10 (42%)	8 (32%)	−10%	X	4 (17%)	4 (16%)	−1%
Hodgkin Lymphoma	X	5 (23%)	8 (25%)	2%	X	11 (50%)	14 (44%)	−6%
Chronic Lymphocytic Leukaemia	X	8 (21%)	8 (33%)	12%	X	9 (24%)	17 (71%)	47%
Mantle Cell Lymphoma	X	11 (41%)	12 (40%)	−1%	X	7 (26%)	8 (27%)	1%
Multiple Myeloma	2 (33%)	6 (21%)	17 (53%)	32%	2 (33%)	15 (54%)	18 (56%)	2%
Myelodysplastic Syndromes	X	7 (29%)	11 (55%)	26%	X	6 (25%)	11 (55%)	30%
Waldenstrom Macroglobulinaemia	X	1 (13%)	0	−13%	X	0	3 (38%)	38%
Bone Sarcomas	4 (14%)	3 (12%)	3 (6%)	−6%	6 (21%)	6 (24%)	8 (17%)	−7%
Early Breast Cancer	39 (47%)	32 (43%)	75 (54%)	11%	42 (51%)	45 (60%)	100 (73%)	13%
Metastatic Breast Cancer	3 (60%)	4 (31%)	29 (42%)	11%	3 (60%)	7 (54%)	31 (45%)	−9%
Gastrointestinal Stromal Tumor	4 (23%)	6 (29%)	8 (26%)	−3%	8 (47%)	11 (52%)	16 (52%)	0
Hepatocellular Carcinoma	X	4 (14%)	17 (32%)	18%	X	17 (61%)	29 (55%)	−6%
Localised Colon Cancer	X	4 (15%)	12 (32%)	17%	X	3 (11%)	16 (42%)	31%
Metastatic Colorectal Cancer	X	21 (67%)	30 (40%)	−27%	X	5 (16%)	31 (41%)	25%
Melanoma	X	0	8 (31%)	31%	X	2 (20%)	9 (35%)	15%
Metastatic Non-Small Cell Lung Cancer	27 (49%)	34 (45%)	71 (42%)	−3%	24 (44%)	34 (45%)	79 (46%)	1%
Non-Small Cell Lung Cancer	X	20 (43%)	36 (32%)	−11%	X	34 (72%)	64 (57%)	−15%
Small-Cell Lung Cancer	X	6 (30%)	11 (18%)	−12%	X	3 (15%)	25 (42%)	27%
Nasopharyngeal Carcinoma	X	3 (21%)	5 (15%)	−6%	X	7 (50%)	12 (35%)	−15%
Oesophageal Cancer	8 (38%)	8 (24%)	15 (43%)	19%	4 (19%)	14 (41%)	25 (71%)	30%
Soft Tissue and Visceral Sarcomas	7 (18%)	7 (27%)	8 (26%)	−1%	12 (32%)	6 (23%)	18 (58%)	35%
Rectal Cancer	X	11 (26%)	9 (47%)	21%	X	28 (65%)	13 (68%)	3%
Gastric Cancer	17 (45%)	18 (39%)	22 (45%)	6%	12 (32%)	16 (35%)	20 (41%)	6%
Renal Cell Carcinoma	11 (27%)	11 (19%)	26 (48%)	29%	18 (44%)	24 (42%)	26 (48%)	6%
Prostate Cancer	11 (39%)	18 (45%)	27 (53%)	8%	8 (29%)	16 (40%)	14 (28%)	−12%
Marginal Zone Lymphomas	X	0	1 (6%)	6%	X	5 (36%)	5 (31%)	−5%
Hereditary Gastrointestinal Cancer	X	X	0	X	X	X	8 (25%)	X
Gestational Trophoblastic Disease	X	X	0	X	X	X	17 (94%)	X
Philadelphia Chromosome Negative Chronic MPNs	X	X	11 (27%)	X	X	X	10 (24%)	X
Peripheral T-cell Lymphomas	X	X	0	X	X	X	4 (36%)	X
Hairy Cell Leukaemia	X	X	5 (12%)	X	X	X	3 (7%)	X
Extranodal Diffuse large B-cell lymphoma (DLBCL) and Primary Mediastinal B-Cell Lymphoma	X	X	2 (5%)	X	X	X	17 (40%)	X
Acute Lymphoblastic Leukaemia	X	X	7 (33%)	X	X	X	11 (52%)	X
Thymic Epithelial Tumors	X	X	0	X	X	X	31 (36%)	X
Penile Cancer	X	X	0	X	X	X	0	X
Leptomeningeal Metastasis	X	X	0	X	X	X	0	X
Brain Metastasis from Solid Tumours	X	X	6 (15%)	X	X	X	5 (13%)	X
Bladder Cancer	X	9 (41%)	9 (19%)	−22%	X	11 (50%)	13 (28%)	−22%
Endometrial Cancer	X	5 (50%)	12 (24%)	−26%	X	5 (50%)	18 (36%)	−14%
Malignant Pleural Mesothelioma	X	1 (4%)	13 (25%)	21%	X	19 (73%)	21 (40%)	−33%

As shown, the cancer area with the highest number of recommendations was pharmacotherapy with a total number of 1603 recommendations of which 209, 452 and 942, were from the first, previous and current versions, respectively. The distribution of levels of evidence (LOE) I and grades of recommendations (GOR) A among different cancer areas is presented in Suppl. Fig. S2.

Among the 41 current guidelines, a total of 1823 recommendations were extracted; 550 (average 30%, median 26%, [25th-75th percentiles (intraquartile range-IQR), 12%–40%]) were classified as LOE I and 794 (average 43%, median 40% [IQR, 28%–52%]) were classified as GOR A. From the previous CPG versions, a total of 885 recommendations were extracted; 284 (average 32%, median 28%, [IQR, 19.50%–41.75%]) were classified as LOE I and 375 as GOR A (average 42%, median 41.5%, [IQR, 24%–51.50%]). These proportions of recommendations among previous and current versions of guidelines are presented in Suppl. Figs. S3 and S4.

There were a total of 11 first CPG versions and 360 recommendations analysed; 133 (average 37%, median 38%, [IQR, 25%–46%]) were classified as LOE I and 139 (average 39%, median 33%, [IQR, 30.50%–45.50%]) as GOR A.

Overall, pharmacotherapy (42%) and supportive therapy (33%) had the highest proportion of LOE I in the current CPG, whereas the lowest proportion of LOE I was found in surgery (16%), genetic alterations (15%), diagnostic (16%) and follow up (10%). Pathology (50%) and diagnostic procedures (50%) had the highest proportion of GOR A recommendations whereas screening (31%) and follow-up (23%) procedures had the lowest proportion of GOR A recommendations. The general distribution of LOE I and GOR A across different cancer fields is fully summarized in Suppl. Tables S3 and S4.

When scrutinizing different cancer types, myelodysplastic syndromes (55%), early breast cancer (54%), multiple myeloma (53%), prostate cancer (53%), renal cell carcinoma (48%) and rectal carcinoma (47%) had the highest proportion of LOE I recommendations. Gestational trophoblastic disease (94%), early breast cancer (76%), oesophageal cancer (71%), rectal (68%), chronic lymphocytic leukemia (CLL; 71%) and rectal cancer (68%) had the highest proportion of GOR A.

### Changes from first/prior to current guidelines

**Figure 2:**
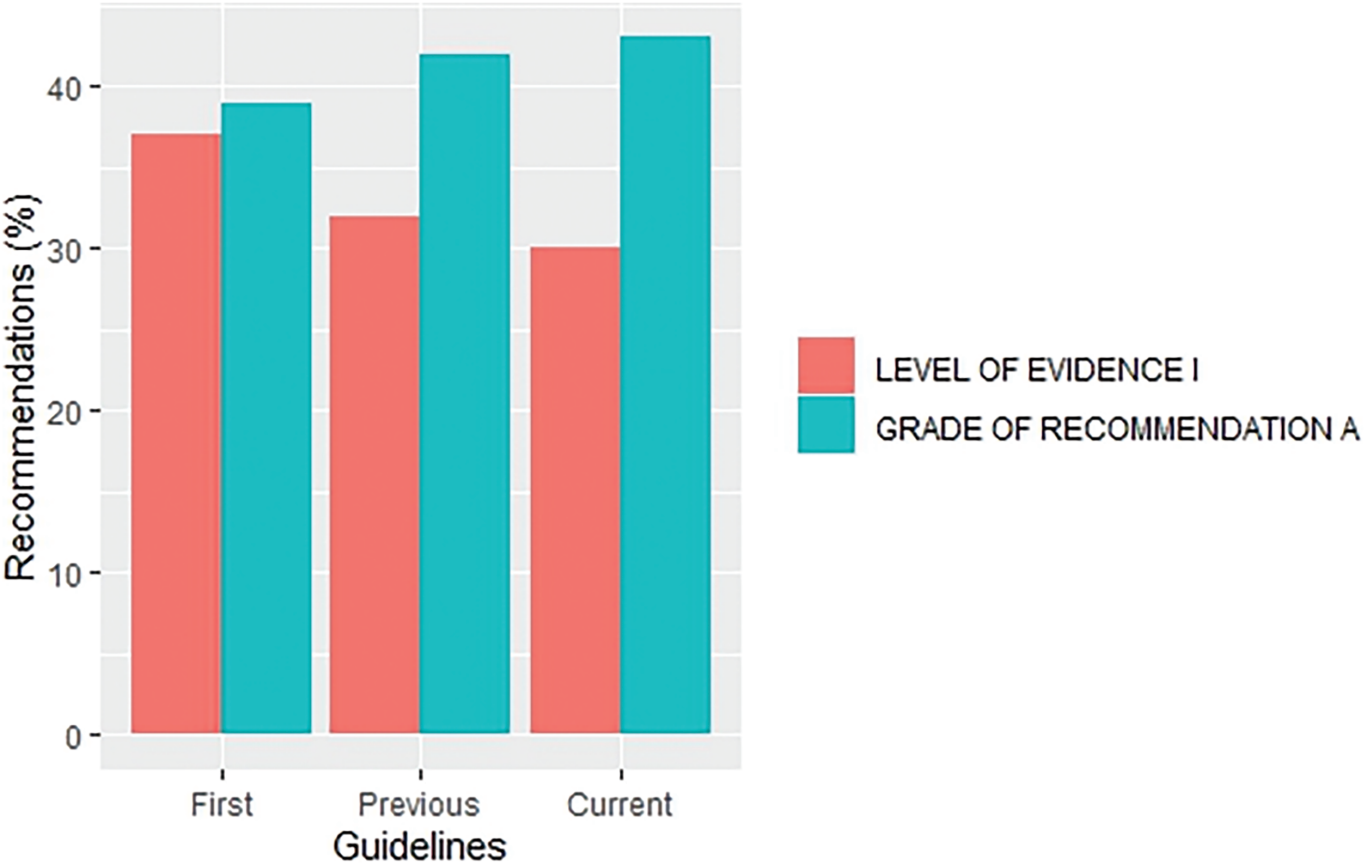
Distribution of recommendations with level of evidence I and grade of recommendation A by first, previous and current versions of overall guidelines.

**Figure 3:**
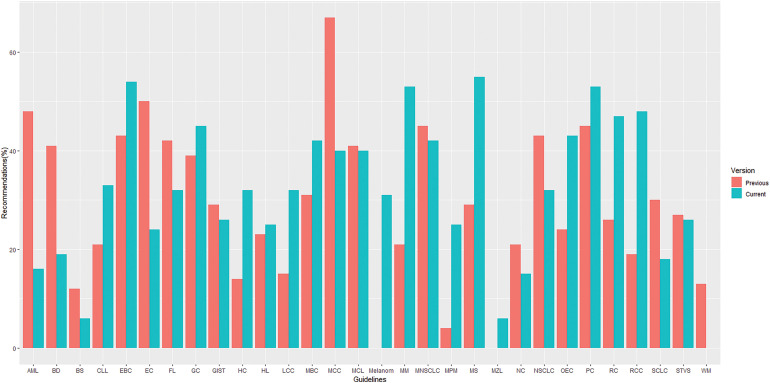
Distribution of recommendations with level of evidence I by topics* in previous and current versions of guidelines. *List of abbreviations in supplementary materials.

**Figure 4:**
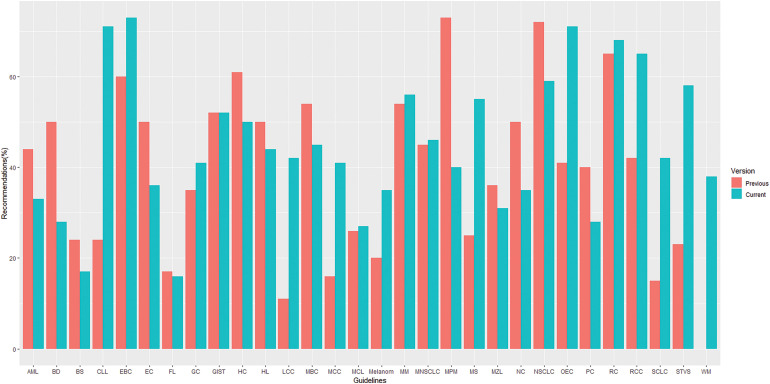
Distribution of recommendations with grade A by topics* in previous and current versions of guidelines. *List of abbreviations in supplementary materials.

The overall proportion of LOE I recommendations across different versions of guidelines demonstrated a declining trend (37% *vs*. 32%. *vs*. 30% for the first, previous and current versions, respectively) whereas, in contrast, GOR A recommendations demonstrated a rising/stable trend (39% *vs*. 42%. *vs*. 43% for the first, previous and current versions, respectively), as shown in Fig. 2. Their distribution by specific cancer types is fully summarized in Figs. 3 and 4.

**Figure 5:**
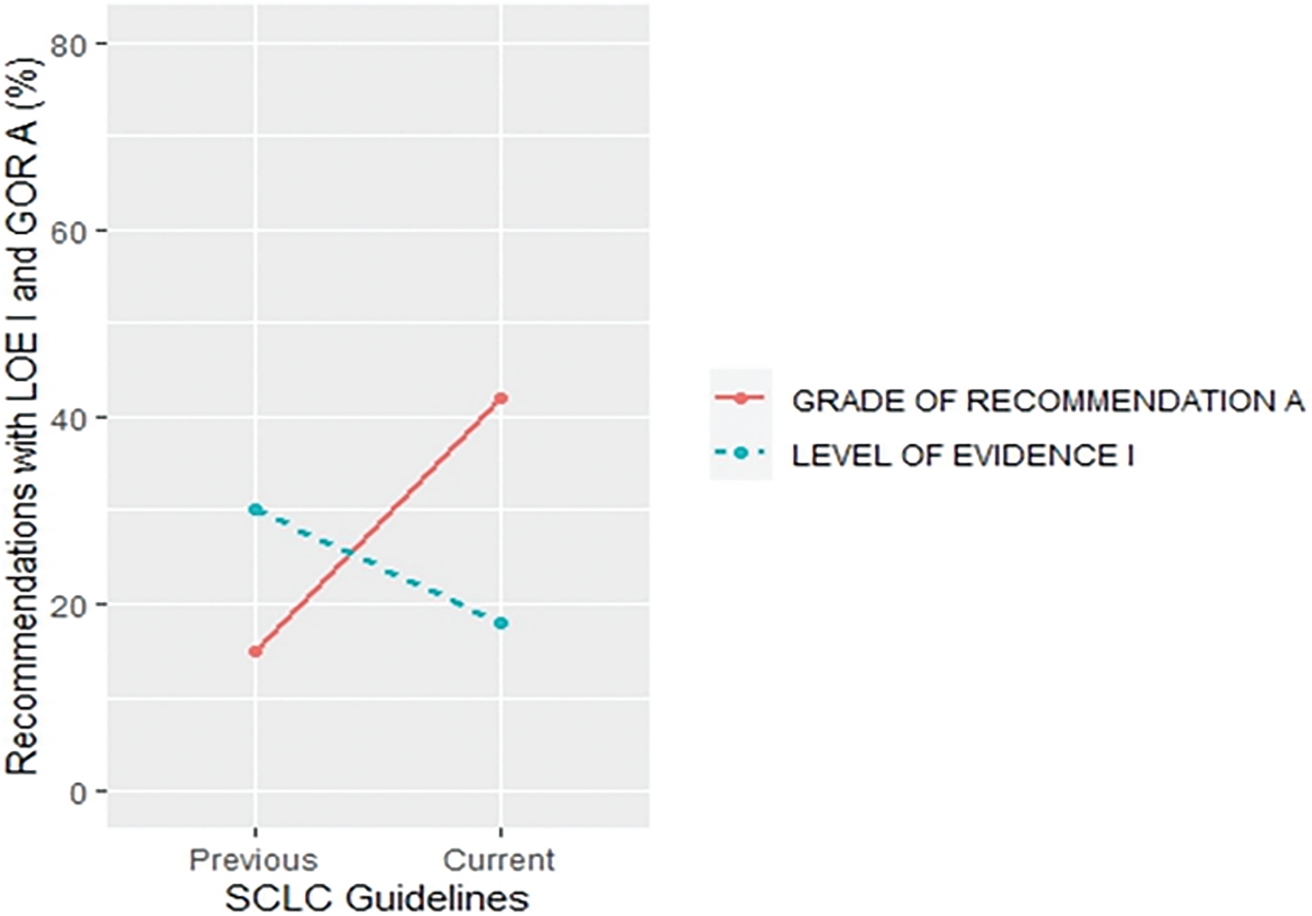
Proportion of recommendations classified as level of evidence I and grade of recommendation A in previous and current small cell lung cancer guideline documents.

**Figure 6:**
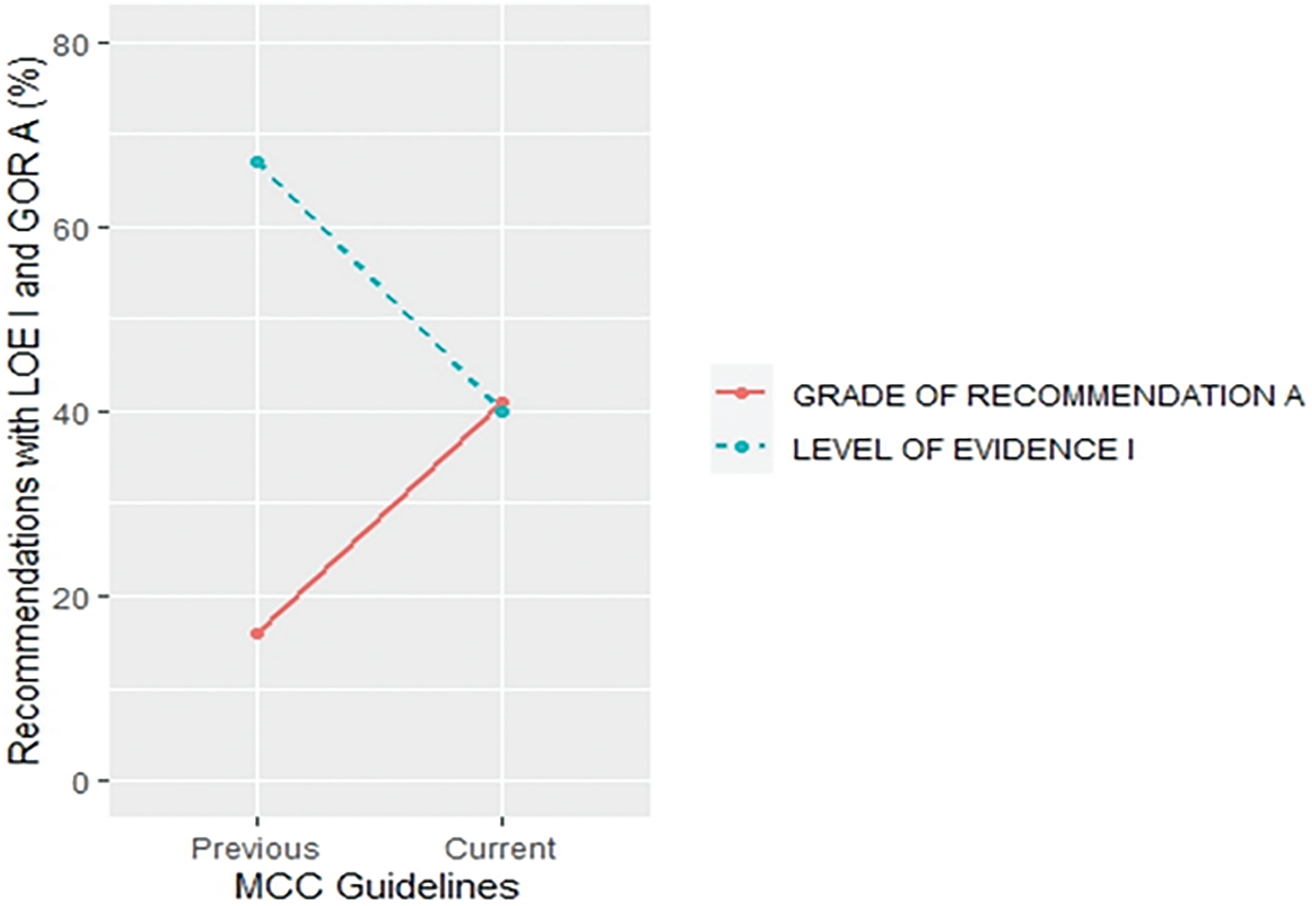
Proportion of recommendations classified as level of evidence I and grade of recommendation A in previous and current metastatic colorectal cancer guideline documents.

More specifically, a trend of a decrease in the proportion of LOE I recommendations and an increase in their GOR A was found in small cell lung cancer (SCLC) (30% *vs*. 18% for LOE I, *p* = 0.430;15% *vs*. 42% for GOR A, *p* = 0.058) (Fig. 5) and metastatic colorectal cancer (MCC) (67% *vs*. 40% for LOE I, *p* = 0.027; 16% *vs*. 41% for GOR A, *p* = 0.019) (Fig. 6) when compared to previous CPG versions.

By contrast, there was a trend of an increase in the proportion of LOE I (4% *vs*. 25%, *p* = 0.047) recommendations and a decrease in GOR A (73% *vs*. 40%, *p* = 0.013) in malignant pleural mesothelioma (Suppl. Fig. S5). In AML, there was a decrease in the number of recommendations with LOE I (48% *vs*. 16%, *p* = 0.008) and their GOR A (44% *vs*. 33%, *p* = 0.563) in the current versions (Suppl. Fig. S6). On the other hand, CLL demonstrated an increase in LOE I (21% *vs*. 33%, *p* = 0.436) and GOR A (24% *vs*. 71%, *p* = 0.001) (Suppl. Fig. S7). In soft tissue and visceral sarcomas, LOE I recommendations increased (18% *vs*. 27%) but GOR A decreased (32% *vs*. 23%) from the first and the previous versions, whereas the opposite effect was found between the previous and the current CPG (27% *vs*. 26% for LOE I, *p* = 1% and 23% *vs*. 58% for GOR A, *p* = 0.017) (Suppl. Fig. S8). In prostate cancer, there was an increase in LOE I recommendations (39%, 45% and 53% for the first, previous and current CPG version, *p* = 0.589, respectively, but with a lower proportion of GOR A in the first (29%) and the current version (28%) when compared to previous CPG version (40%) (*p* = 0.299) (Suppl. Fig. S9). In addition, the proportion of GOR A recommendations based on a higher level of evidence such as randomized trials (LOE I–II) shows a decrease (71% *vs*. 63%, *p* = 0.009) while recommendations based on a lower level of evidence (LOE III–V) shows an increase (29% *vs*. 37%, *p* = 0.01) between previous and current version.

## Discussion

This is the first study to analyse the quality of evidence and the supporting grades of recommendation of the ESMO CPG. As presented, the ESMO has largely increased the number of its recommendations from 885 in the previous version to 1823 in the current version which is an increase of 106% in the period of 2012–2022. A similar observation was also shown during analysis of the NCCN guidelines where the number of recommendations increased by 77% in the period from 2010 to 2019 [13].

When comparing the current ESMO CPG with their prior version, there was a decrease (−10%, *p* < 0.001) in the proportion of recommendations associated with randomized clinical trials (RCTs) (Level I–II). These results demonstrate that efforts over the past decades to simplify and facilitate clinical trials have not yet translated into evidence better supported by RCTs. On the other hand, studies without a control group, case reports, and expert opinions, that is, those that are associated with Level V evidence, have shown an increase of 5% from previous to current ESMO CPG. This observation is worrisome since these types of evidence are of lower quality and may be prone to conflicts of interest. Overall, the proportion of recommendations with the highest level of evidence (LOE I) demonstrated a slight decrease in their current versions when compared to previous versions (32% *vs*. 30%, *p* = 0.331). Also, a large proportion (49%) of recommendations in the current guidelines had no supporting data from RCT (Level III–V). It should be pointed out that evidence provided by non-RCT can provide valuable information to practicing clinicians; however, only RCT can enable true comparisons between the different arms regardless of intervention Approximately one-third (30%) of the current ESMO CPG recommendations are supported by LOE I, which is in contrast with NCCN guidelines (category 1, 7%) [14]. However, complete comparisons of ESMO LOE I and NCCN categories of evidence and consensus may not be suitable since those definitions of the categories are different in form the NCCN definition of category 1 evidence and consensus is much more strict (typically requiring multiple randomized clinical trials or meta-analysis) compared to ESMO level of evidence I.

However, there was a substantial variability by topics and cancer types when considering the number of recommendations associated with the LOE I and GOR A recommendations. A high proportion of recommendations considering pharmacotherapy (44%) has been noticed. One possible explanation may be the strict regulatory requirements by the European Medicines Agency (EMA) and the US Food and Drug Administration (FDA). Additionally, the presence of accessible funding provided by pharmaceutical companies in order to conduct such trials can also be a contributing factor. Conversely, there was a relatively low proportion of LOE I in the surgery setting (16%) which may be surprising when considering that surgery is often the only curable option in some clinical scenarios. However, conducting RCT in surgery has its issues, such as imprecise definitions of interventions and outcomes, inadequate control arms, randomization and recruitment issues, long surgery learning curves, and rare or urgent conditions [15].

Similarly, as noticed during NCCN guidelines analysis [8], the proportion of recommendations regarding the follow-up (Level I 10%) was also based on the lower proportion of high-quality evidence when compared to other settings.

There was an increase in poor quality evidence (Level V) in the current guidelines when compared to the previous ones for acute myeloid leukemia (AML, 14%, *p* = 0.013), bone sarcoma (13%, *p* = 0.284), NSCLC (15%, *p* = 0.011), nasopharyngeal cancer (15%, *p* = 0.319) and metastatic breast cancer (13%, *p* = 0.37) (Suppl. Table S3). However, there was a huge increase in multiple myeloma (32%, *p* = 0.024), myelodysplastic syndrome (26%, *p* = 0.153), melanoma (31%, *p* = 0.123) and renal cell carcinoma (29%, *p* = 0.003) in LOE I recommendations while metastatic colorectal cancer (−27%, *p* = 0.027), AML (−32%, *p* = 0.008) and endometrial cancer (−26%, *p* = 0.2) have shown a decrease in the proportion of LOE I recommendations in the current when compared to previous guidelines. Therefore, more emphasis should be attached to future research in order to make poorer evidence substantially rarer in the malignant setting as it has been made in the setting of localized colon cancer where the proportion flow-quality evidence (Level IV and V) has been decreased by 38% (Suppl. Table S3).

There was an increase in the recommendations with the most certainty to do something (Grade A +1%, *p* = 0.645) while the recommendations associated with the lowest certainty have not been meaningfully increased (Grade C +2%, *p* = 0.131) in the current when compared to their previous version (Suppl. Table S4). Data on the proportion of GOR A recommendations based on a higher level of evidence such as randomized trials (LOE I–II) shows a decrease (71% *vs*. 63%, *p* = 0.009) while the proportion of GOR A based on a lower level of evidence (LOE III–V) shows an increase (29% *vs*. 37%, *p* = 0.01) between previous and current version. More interestingly, changes from the first to previous and current versions show that while the percentage of LOE I goes down, the confidence that the recommendations are correct goes up (GOR A) (Fig. 2). This change was found especially in SCLC (Fig. 5) and MCC (Fig. 6) guidelines between the previous and current versions. In comparison to previous ESMO CPG, there was a significant increase in the proportion of uncertain recommendations (Grade C) in follicular lymphoma (12%, *p* = 0.37) and metastatic breast cancer (12%, *p* = 0.434) guidelines, while a substantial decrease in uncertain recommendations (Grade C) has been seen in myelodysplastic syndrome (−24%, *p* = 0.093), SCLC (−22%, *p* = 0.133) and soft tissue and visceral sarcoma (−39%, *p* = 0.001) CPG (Suppl. Table S4).

More interestingly, there were significant discrepancies in SCLC (Fig. 5), metastatic colorectal (Fig. 6), malignant pleural mesothelioma (Suppl. Fig. S5), AML (Suppl. Fig. S6), CLL (Suppl. Fig. S7), soft tissue and visceral sarcoma (Suppl. Fig. S8) and prostate guidelines (Suppl. Fig. S9). These discrepancies were present in the form of unexpected changes in the proportion of LOE I and GOR A through different versions of guidelines. One possible reason could represent significant advances in the particular cancer field which could have made prior CPG recommendations obsolete so they were not included in the current version. However, these recommendations were not replaced with the same quality of evidence and, surprisingly, the grades of recommendations were not in line with the change in the quality of evidence.

Limitations of this analysis are the lack of independent assessment of all classifications included in this paper. However, ESMO CPG represents the state-of-the-art guidelines regarding the treatment of malignant diseases in Europe and, as such, we have assumed that all classifications have been made correctly. Additionally, we have assessed only general recommendations within different cancer fields and cancer types, not those particularly related to every clinical condition related to specific cancers. One of the limitations is that the study results may be influenced by the time lag between the issuance of different versions of the guidelines. * This is particularly evident when comparing cancer settings with low to high incidence.To enhance the strength of evidence underpinning CPGs and elevate their reliability for practicing clinicians, future oncology research should prioritize well-designed randomized controlled clinical trials.

Nevertheless, despite these limitations, the presented analysis demonstrates that there is still a lot of space for conducting high quality research which could further improve the ESMO CPG. More well-designed RCT are needed to improve clinical outcomes in different cancers, especially when considering a global rise in cancer incidence worldwide.

## Supplementary Materials

Figure S1 Number of recommendations in First, Previous and Current Guideline Version

Figure S2 Distribution of Recommendations with Grade A and Level of Evidence I by Type of Recommendation

Figure S3 Proportion of Recommendations with Level of Evidence I Among Different Versions of Guidelines

Figure S4 Proportion of Recommendations with Grade A Among Different Versions of Guidelines

Figure S5 Proportion of Recommendations classified as Level of Evidence I and Grade of Recommendation A in Previous and Current Malignant Pleural Mesothelioma Guideline Documents

Figure S6 Proportion of Recommendations classified as Level of Evidence I and Grade of Recommendation A in Previous and Current Acute Myeloid Leukaemia Guideline Documents

Figure S7 Proportion of Recommendations classified as Level of Evidence I and Grade of Recommendation A in Previous and Current Chronic Lymphocytic Leukaemia Guideline Documents

Figure S8 Proportion of Recommendations classified as Level of Evidence I and Grade of Recommendation A in First, Previous and Current Soft Tissue and Visceral Sarcomas Guideline Documents

Figure S9 Proportion of Recommendations classified as Level of Evidence I and Grade of Recommendation A in First, Previous and Current Prostate Cancer Guideline Documents

## Data Availability

The data that support the findings of this study are available from the corresponding author upon reasonable request.

## Supplementary Materials

The supplementary material is available online at https://doi.org/10.32604/or.2024.048948.
