# Supplementary figures and images for: Levels of evidence and grades of recommendation supporting European society for medical oncology clinical practice guidelines
Source: Oncol Res. 2024 Apr 23;32(5):807–15. doi: 10.32604/or.2024.048948 (PMC11055998; doi:10.32604/or.2024.048948)

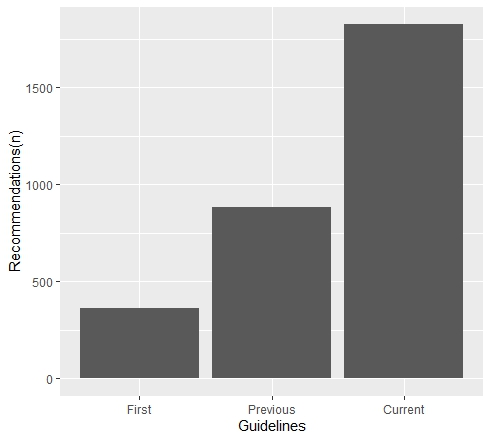

Supplement: Figure S1 [file OncolRes-32-48948-s001.tiff]

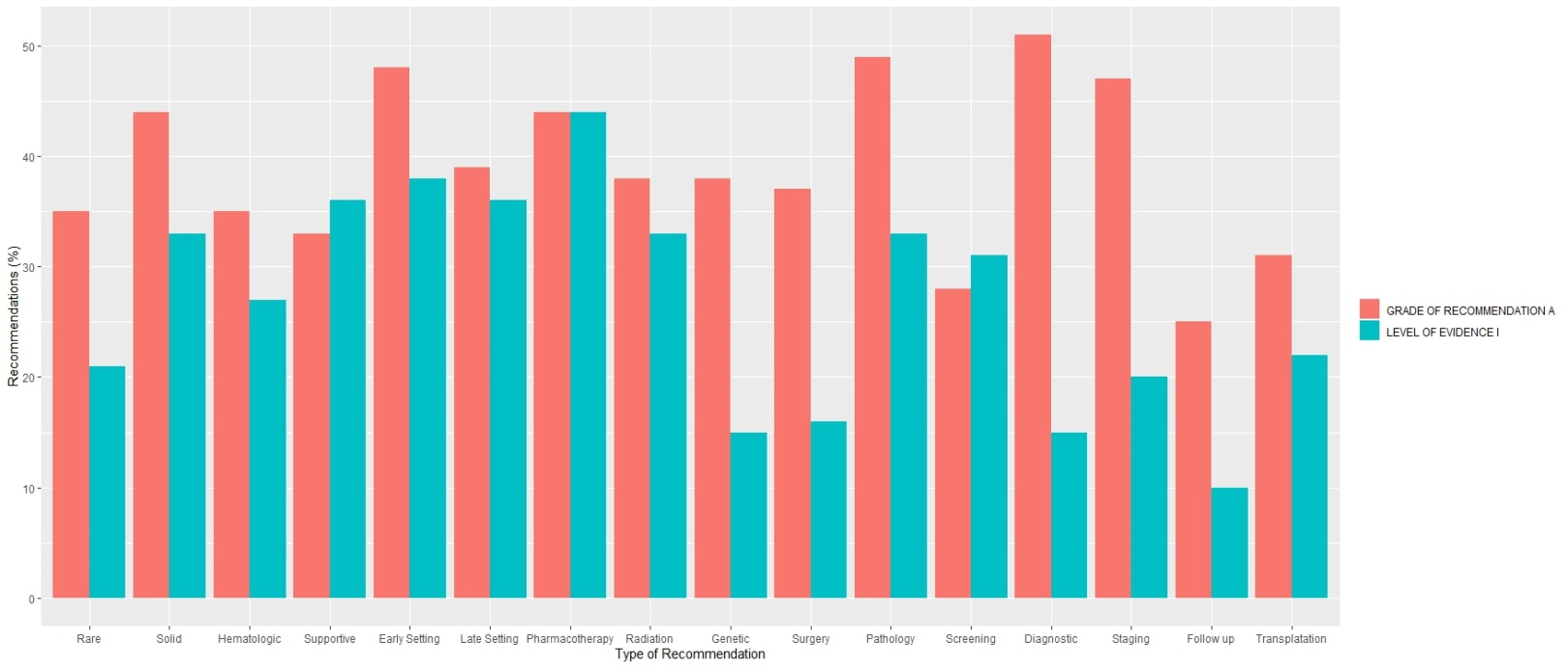

Supplement: Figure S2 [file OncolRes-32-48948-s002.tiff]

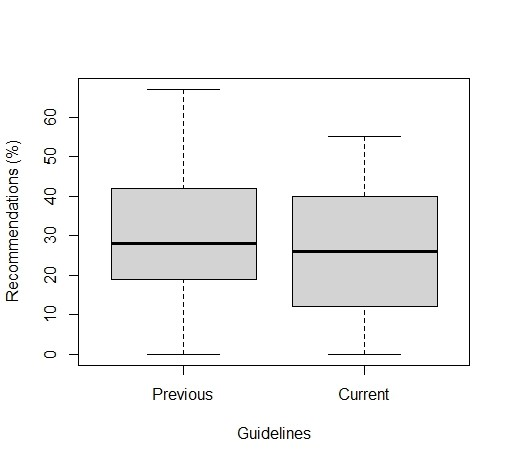

Supplement: Figure S3 [file OncolRes-32-48948-s003.tiff]

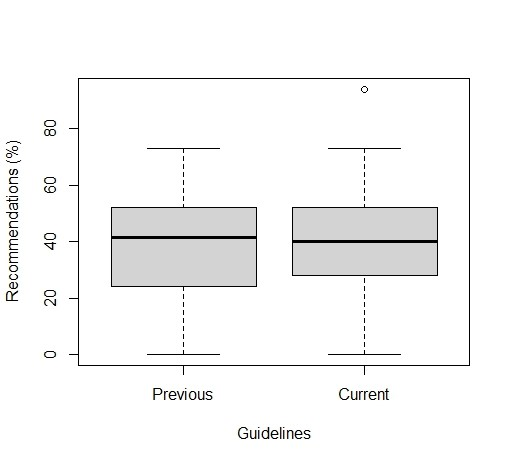

Supplement: Figure S4 [file OncolRes-32-48948-s004.tiff]

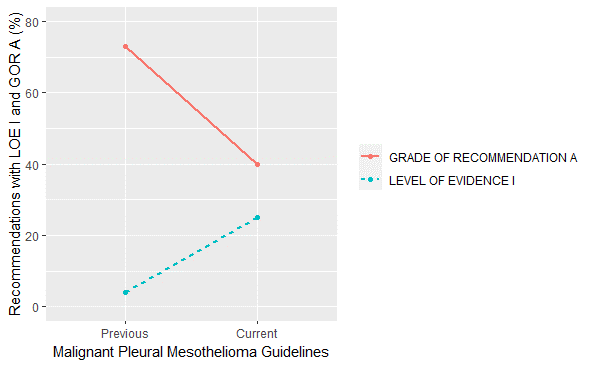

Supplement: Figure S5 [file OncolRes-32-48948-s005.tiff]

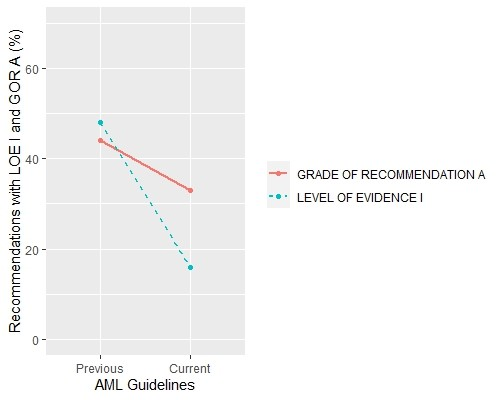

Supplement: Figure S6 [file OncolRes-32-48948-s006.tiff]

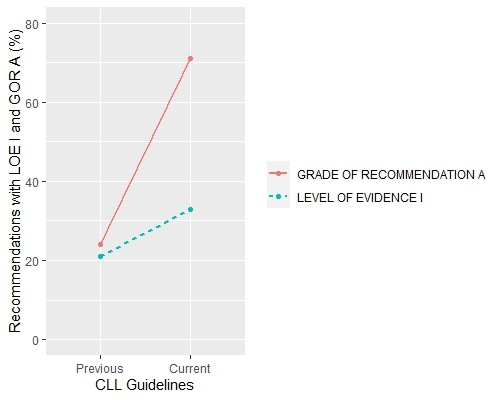

Supplement: Figure S7 [file OncolRes-32-48948-s007.tiff]

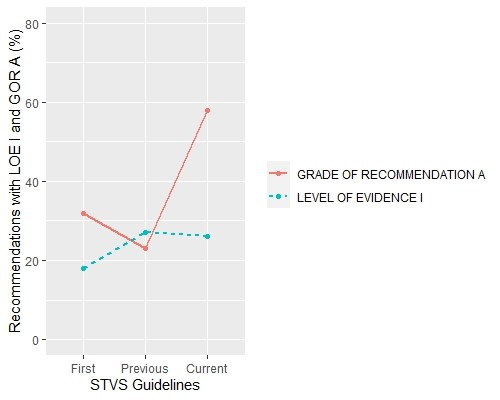

Supplement: Figure S8 [file OncolRes-32-48948-s008.tiff]

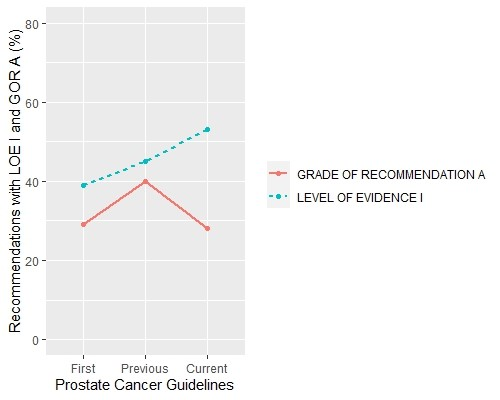

Supplement: Figure S9 [file OncolRes-32-48948-s009.tiff]
