# Supplementary material for: Levels of evidence and grades of recommendation supporting European society for medical oncology clinical practice guidelines
Source: Oncol Res. 2024 Apr 23;32(5):807–15. doi: 10.32604/or.2024.048948 (PMC11055998; doi:10.32604/or.2024.048948)
Supplement: Supplementary file 10 [file OncolRes-32-48948-s001.docx]

**Table S1: ESMO clinical practice guidelines and associated level of evidence**

| **n (%)** | **II**  **Previous** | **II**  **Current** | **Difference**  **(current vs previous)** | **III**  **Previous** | **III**  **Current** | **Difference**  **(current vs previous)** | **IV**  **Previous** | **IV**  **Current** | **Difference**  **(current vs previous)** | **V**  **Previous** | **V**  **Current** | **Difference**  **(current vs**  **previous)** |
| --- | --- | --- | --- | --- | --- | --- | --- | --- | --- | --- | --- | --- |
| **All** | 208(23%) | 297(16%) | -7% | 220(25%) | 447(24%) | -1% | 128(14%) | 341(19%) | 5% | 47(5%) | 190(10%) | 5% |
| **Acute**  **Myeloid Leukaemia** | 11(48%) | 22(43%) | -5% | 1(4%) | 8(16%) | 12% | 0 | 6(12%) | 12% | 0 | 7(14%) | 14% |
| **Follicular Lymphoma** | 7(29%) | 5(20%) | -9% | 1(4%) | 3(12%) | 8% | 5(21%) | 7(28%) | 7% | 1(4%) | 2(8%) | 4% |
| **Hodgkin Lymphoma** | 5(23%) | 6(19%) | -4% | 6(27%) | 14(44%) | 17% | 4(18%) | 13(3%) | -15% | 2(9%) | 3(9%) | 0 |
| **Chronic Lymphocytic Leukaemia** | 0 | 2(8%) | 8% | 22(58%) | 6(25%) | -33% | 6(16%) | 5(21%) | 5% | 2(5%) | 3(13%) | 8% |
| **Mantle**  **Cell Lymphoma** | 5(19%) | 5(17%) | -2% | 3(11%) | 5(17%) | 6% | 7(26%) | 7(23%) | -3% | 1(4%) | 1(3%) | -1% |
| **Multiple Myeloma** | 20(71%) | 11(34%) | -37% | 2(7%) | 3 (9%) | 2% | 0 | 1 (3%) | 3% | 0 | 0 | 0 |
| **Myelodysplastic**  **Syndromes** | 3(13%) | 6(30%) | 17% | 5(21%) | 3(15%) | -6% | 9(38%) | 0 | -38% | 0 | 0 | 0 |
| **Waldenstrom**  **Macroglobulinaemia** | 3(38%) | 0 | -38% | 4(50%) | 4(50%) | 0 | 0 | 3(38%) | 38% | 0 | 1(13%) | 13% |
| **Bone Sarcomas** | 3(12%) | 3(6%) | -6% | 7(28%) | 10(21%) | -7% | 10(40%) | 22(46%) | 6% | 2(8%) | 10(21%) | 13% |
| **Early**  **Breast Cancer** | 9(12%) | 19(14%) | 2% | 17(23%) | 13(9%) | -14% | 5(7%) | 5(4%) | -3% | 12(16%) | 26(19%) | 3% |
| **Metastatic**  **Breast Cancer** | 5(39%) | 19(28%) | -11% | 4(31%) | 9(13%) | -18% | 0 | 3(4%) | 4% | 0 | 9(13%) | 13% |
| **Gastrointestinal Stromal Tumor** | 2(9%) | 3(10%) | 1% | 6(29%) | 10(32%) | 3% | 7(33%) | 8(26%) | -7% | 0 | 2(6%) | 6% |
| **Hepatocellular Carcinoma** | 4(14%) | 3(6%) | -8% | 20(71%) | 22(41%) | -30% | 1(4%) | 11(21%) | 17% | 0 | 0 | 0 |
| **Localised**  **Colon Cancer** | 4(15%) | 9(24%) | 9% | 5(19%) | 10(26%) | 7% | 10(37%) | 5(13%) | -24% | 5(19%) | 2(5%) | -14% |
| **Metastatic Colorectal Cancer** | 5(16%) | 15(20%) | 4% | 0 | 17(23%) | 23% | 6(19%) | 9(12%) | -7% | 0 | 4(5%) | 5% |
| **Melanoma** | 7(70%) | 6(23%) | -47% | 3(30%) | 9(35%) | 5% | 0 | 2(8%) | 8% | 0 | 1(4%) | 4% |
| **Metastatic Non-Small Cell**  **Lung Cancer** | 23(31%) | 26(15%) | -16% | 12(16%) | 43(25%) | 9% | 6(8%) | 28(16%) | 8% | 0 | 3(2%) | 2% |
| **Non-Small Cell**  **Lung Cancer** | 5(11%) | 8(7%) | -4% | 21(45%) | 44(39%) | -6% | 1(2%) | 8(7%) | 5% | 0 | 17(15%) | 15% |
| **Small-Cell**  **Lung Cancer** | 7(35%) | 17(28%) | -7% | 2(10%) | 8(13%) | 3% | 0 | 11(18%) | 18% | 5(25%) | 13(22%) | -3% |
| **Nasopharyngeal Carcinoma** | 4(29%) | 9(27%) | -2% | 7(50%) | 12(35%) | -15% | 0 | 3(9%) | 9% | 0 | 5(15%) | 15% |
| **Oesophageal Cancer** | 9(27%) | 6(17%) | -10% | 10(29%) | 7(20%) | -9% | 5(15%) | 6(17%) | 2% | 2(6%) | 2(6%) | 0 |
| **Soft Tissue and Visceral Sarcomas** | 8(31%) | 6(19%) | -12% | 3(12%) | 8(26%) | 14% | 8(31%) | 8(26%) | -5% | 0 | 1(3%) | 3% |
| **Rectal Cancer** | 9(21%) | 0 | -21% | 11(26%) | 7(37%) | 11% | 10(23%) | 1(5%) | -18% | 2(5%) | 2(11%) | 6% |
| **Gastric Cancer** | 4(9%) | 9(18%) | 9% | 10(22%) | 4(8%) | -14% | 10(22%) | 7(14%) | -8% | 4(9%) | 7(14%) | 5% |
| **Renal**  **Cell Carcinoma** | 20(35%) | 12(22%) | -13% | 16(28%) | 9(17%) | -31% | 6(11%) | 7(13%) | 2% | 4(7%) | 0 | -7% |
| **Prostate Cancer** | 6(15%) | 5(10%) | -5% | 9(23%) | 15(29%) | 6% | 4(10%) | 4(8%) | -2% | 3(8%) | 0 | -8% |
| **Marginal Zone Lymphomas** | 2(14%) | 2 (12%) | -2% | 6(43%) | 6 (37%) | -6% | 6(43%) | 7 (44%) | 1% | 0 | 0 | 0 |
| **Hereditary Gastrointestinal Cancer** | X | 1(3%) | X | X | 16(50%) | X | X | 11(34%) | X | X | 4(12%) | X |
| **Gestational Trophoblastic Disease** | X | 2(11%) | X | X | 0 | X | X | 16(89%) | X | X | 0 | X |
| **Philadelphia Chromosome Negative Chronic MPNs** | X | 3(7%) | X | X | 13(32%) | X | X | 12(29%) | X | X | 2(5%) | X |
| **Peripheral T-cell Lymphomas** | X | 0 | X | X | 8(73%) | X | X | 1(9%) | X | X | 2(18%) | X |
| **Hairy Cell Leukaemia** | X | 8(19%) | X | X | 6(14%) | X | X | 12(29%) | X | X | 11(26%) | X |
| **Extranodal DLBCLand Primary Mediastinal B-Cell Lymphoma** | X | 6(14%) | X | X | 26(62%) | X | X | 6(14%) | X | X | 2(5%) | X |
| **Acute Lymphoblastic Leukaemia** | X | 1 (5%) | X | X | 6(29%) | X | X | 5(24%) | X | X | 2(9%) | X |
| **Thymic Epithelial Tumors** | X | 1(1%) | X | X | 21(25%) | X | X | 36(42%) | X | X | 27(32%) | X |
| **Penile Cancer** | X | 0 | X | X | 5(100%) | X | X | 3(15%) | X | X | 0 | X |
| **Leptomeningeal Metastasis** | X | 1(33%) | X | X | 1(33%) | X | X | 1(33%) | X | X | 0 | X |
| **Brain Metastasis from Solid Tumours** | X | 6 (15%) | X | X | 6 (15%) | X | X | 14 (36%) | X | X | 8(20%) | X |
| **Bladder Cancer** | 5(23%) | 8(17%) | -6% | 7(32%) | 9(19%) | -13% | 0 | 21(45%) | 45% | 1(4%) | 0 | -4% |
| **Endometrial Cancer** | 2(20%) | 11(22%) | 2% | 2(20%) | 10(20%) | 0 | 1(10%) | 13(26%) | 16% | 0 | 4(8%) | 8% |
| **Malignant Pleural Mesothelioma** | 13(50%) | 15(29%) | -21% | 4(15%) | 10(19%) | 4% | 7(27%) | 8(15%) | -12% | 1(4%) | 6(11%) | 7% |

**Table S2**

**ESMO clinical practice guidelines and associated grade of recommendation**

| **n (%)** | **B**  **Previous** | **B**  **Current** | **Difference**  **(currentvs previous)** | **C**  **Previous** | **C**  **Current** | **Difference**  **(current vs previous)** | **D**  **Previous** | **D**  **Current** | **Difference**  **(current vs previous** | **E**  **Previous** | **E**  **Current** | **Difference (current vs previous)** |
| --- | --- | --- | --- | --- | --- | --- | --- | --- | --- | --- | --- | --- |
| **All** | 372(42%) | 672(37%) | -5% | 104(12%) | 254(14%) | 2% | 32(4%) | 67(4%) | 0 | 2(0,2%) | 34(2%) | 1,8% |
| **Acute**  **Myeloid Leukaemia** | 5(22%) | 20(39%) | 17% | 6(26%) | 11(22%) | -4% | 2(9%) | 2(4%) | -5% | 0 | 1(2%) | 2% |
| **Follicular Lymphoma** | 15(63%) | 15(60%) | -3% | 1(4%) | 4(16%) | 12% | 4(17%) | 2(8%) | -9% | 0 | 0 | 0 |
| **Hodgkin Lymphoma** | 9(41%) | 16(50%) | 9% | 2(9%) | 2(6%) | -3% | 0 | 0 | 0 | 0 | 0 | 0 |
| **Chronic**  **Lymphocytic Leukaemia** | 24(63%) | 2(8%) | -55% | 5(13%) | 2(8%) | -5% | 0 | 1(4)% | 4% | 0 | 2(8%) | 8% |
| **Mantle Cell Lymphoma** | 13(48%) | 14(47%) | -1% | 2(7%) | 3(10%) | 3% | 5(19%) | 5(17%) | -2% | 0 | 0 | 0 |
| **Multiple Myeloma** | 9(32%) | 11(34%) | 2% | 4(14%) | 3 (9%) | -5% | 0 | 0 | 0 | 0 | 0 | 0 |
| **Myelodysplastic Syndromes** | 11(46%) | 8(40%) | -6% | 7(29%) | 1(5%) | -24% | 0 | 0 | 0 | 0 | 0 | 0 |
| **Waldenstrom**  **Macroglobulinaemia** | 8(100%) | 2(25%) | -75% | 0 | 2(25%) | 25% | 0 | 1(13%) | 13% | 0 | 0 | 0 |
| **Bone Sarcomas** | 14(56%) | 34(71%) | 15% | 4(16%) | 6(13%) | -3% | 1(4%) | 0 | -4% | 0 | 0 | 0 |
| **Early Breast Cancer** | 21(28%) | 22(16%) | -12% | 4(5%) | 4(3%) | -2% | 4(5%) | 6(4%) | -1% | 1(1%) | 6(4%) | 3% |
| **Metastatic Breast Cancer** | 6(46%) | 23(33%) | -13% | 0 | 8(12%) | 12% | 0 | 2(3%) | 3% | 0 | 0 | 0 |
| **Gastrointestinal**  **Stromal Tumor** | 5(24%) | 8(26%) | 2% | 3(14%) | 5(16%) | 2% | 2(9%) | 2(7%) | -3% | 0 | 0 | 0 |
| **Hepatocellular Carcinoma** | 10(36%) | 16(30%) | -6% | 1(4%) | 4(7%) | 3% | 0 | 1(2%) | 2% | 0 | 3(6%) | 6% |
| **Localised Colon Cancer** | 13(48%) | 15(40%) | -8% | 6(22%) | 7(18%) | -4% | 5(19%) | 0 | -19% | 0 | 0 | 0 |
| **Metastatic Colorectal Cancer** | 27(84%) | 28(37%) | -47% | 0 | 7(9%) | 9% | 0 | 5(7%) | 7% | 0 | 4(5%) | 5% |
| **Melanoma** | 5(50%) | 10(39%) | -11% | 3(30%) | 4(15%) | -15% | 0 | 2(8%) | 8% | 0 | 1(4%) | 4% |
| **Metastatic Non-Small Cell**  **Lung Cancer** | 34(45%) | 66(39%) | -6% | 7(9%) | 26(15%) | 6% | 0 | 0 | 0 | 0 | 0 | 0 |
| **Non-Small Cell**  **Lung Cancer** | 11(23%) | 36(32%) | 9% | 2(4%) | 11(10%) | 6% | 0 | 0 | 0 | 0 | 2 (2%) | 2% |
| **Small-Cell Lung Cancer** | 7(35%) | 16(27%) | -8% | 10(50%) | 17(28%) | -22% | 0 | 1(2%) | 2% | 0 | 1(2%) | 2% |
| **Nasopharyngeal Carcinoma** | 6(43%) | 20(59%) | 16% | 0 | 2(6%) | 6% | 0 | 0 | 0 | 1(7%) | 0 | -7% |
| **Oesophageal Cancer** | 15(44%) | 9(26%) | 18% | 4(12%) | 1(3%) | -9% | 1(3%) | 0 | -3% | 0 | 0 | 0 |
| **Soft Tissue and Visceral Sarcomas** | 7(27%) | 10(32%) | 5% | 11(42%) | 1(3%) | -39% | 2(8%) | 2(7%) | -1% | 0 | 0 | 0 |
| **Rectal Cancer** | 8(19%) | 4(21%) | 2% | 4(9%) | 1(5%) | -4% | 3(7%) | 1(5%) | -2% | 0 | 0 | 0 |
| **Gastric Cancer** | 24(52%) | 18(37%) | -15% | 6(13%) | 6(12%) | -1% | 0 | 4(8%) | 8% | 0 | 1(2%) | 2% |
| **Renal Cell Carcinoma** | 24(42%) | 15(28%) | -14% | 6(11%) | 7(13%) | 2% | 3(5%) | 6(11%) | 6% | 0 | 0 | 0 |
| **Prostate Cancer** | 22(55%) | 27(53%) | -2% | 2(5%) | 5(10%) | 5% | 0 | 3(6%) | 6% | 0 | 2(4%) | 4% |
| **Marginal Zone Lymphomas** | 7 (50%) | 9 (56%) | 6% | 1(7%) | 2(12%) | -5% | 1(7%) | 0 | -7% | 0 | 0 | 0 |
| **Hereditary Gastrointestinal Cancer** | X | 12(37%) | X | X | 12(37%) | X | X | 0 | X | X | 0 | X |
| **Gestational Trophoblastic Disease** | X | 1(7%) | X | X | 0 | X | X | 0 | X | X | 0 | X |
| **Philadelphia Chromosome Negative Chronic MPNs** | X | 21(51%) | X | X | 6(15%) | X | X | 4(10%) | X | X | 0 | X |
| **Peripheral T-cell Lymphomas** | X | 5(45%) | X | X | 2(18%) | X | X | 0 | X | X | 0 | X |
| **Hairy Cell Leukaemia** | X | 30(71%) | X | X | 9(21%) | X | X | 0 | X | X | 0 | X |
| **Extranodal DLBCLand Primary Mediastinal B-Cell Lymphoma** | X | 18(43%) | X | X | 7(17%) | X | X | 0 | X | X | 0 | X |
| **Acute Lymphoblastic Leukaemia** | X | 7(33%) | X | X | 2(9%) | X | X | 1(5%) | X | X | 0 | X |
| **Thymic Epithelial Tumors** | X | 25(29%) | X | X | 19(22%) | X | X | 5(6%) | X | X | 5(6%) | X |
| **Penile Cancer** | X | 0 | X | X | 5(100%) | X | X | 0 | X | X | 0 | X |
| **Leptomeningeal Metastasis** | X | 2(67%) | X | X | 1(33%) | X | X | 0 | X | X | 0 | X |
| **Brain Metastasis from Solid Tumours** | X | 28(72%) | X | X | 5(13%) | X | X | 0 | X | X | 1(3%) | X |
| **Bladder Cancer** | 9(41%) | 23(49%) | 8% | 2(9%) | 10(21%) | 12% | 0 | 1(2%) | 2% | 0 | 0 | 0 |
| **Endometrial Cancer** | X | 11(22%) | 22% | 5(50%) | 16(32%) | 18% | 0 | 3(6%) | 6% | 0 | 0 | 0 |
| **Malignant Pleural Mesothelioma** | 5(19%) | 13(25%) | 6% | 2(8%) | 8(15%) | 7% | 0 | 7(13%) | 13% | 0 | 3(6%) | 6% |

**Table S3**

**Types of recommendation and associated level of evidence**

| **TYPE**  **OF RECOMMENDATION** | **I**  **First**  **n (%)** | **I**  **Previous**  **n (%)** | **I**  **Current**  **n (%)** | **I I**  **First**  **n (%)** | **II**  **Previous**  **n (%)** | **II**  **Current**  **n (%)** | **III**  **First**  **n (%)** | **III**  **Previous**  **n (%)** | **III**  **Current**  **n (%)** | **IV**  **First**  **n (%)** | **IV**  **Previous**  **n (%)** | **IV**  **Current**  **n (%)** | **V**  **First**  **n (%)** | **V**  **Previous**  **n (%)** | **V**  **Current**  **n (%)** |
| --- | --- | --- | --- | --- | --- | --- | --- | --- | --- | --- | --- | --- | --- | --- | --- |
| **RARE CANCER** | 17(19) | 81(28) | 121(18) | 14(16) | 74(26) | 109(16) | 34(38) | 62(22) | 175(26) | 15(17) | 62(21) | 179(27) | 9(10) | 13(4) | 90(13) |
| **SOLID CANCER** | 131(37) | 225(33) | 452(32) | 79(22) | 154(22) | 220(15) | 82(23) | 176(25) | 336(23) | 39(11) | 97(14) | 268(19) | 23(6) | 41(6) | 153(11) |
| **HAEMATOLOGY CANCER** | 2(33) | 59(30) | 98(25) | 3(50) | 54(28) | 77(19) | 1(17) | 44(23) | 111(28) | 0 | 31(16) | 73(18) | 0 | 6(3) | 37(9) |
| **SUPPORTIVE THERAPY** | 18(49) | 16(29) | 27(36) | 11(30) | 23(42) | 14(19) | 6(16) | 10(18) | 18(24) | 1(3) | 5(9) | 13(17) | 1(3) | 1(2) | 3(4) |
| **EARLY SETTING** | 64(40) | 119(41) | 214(35) | 32(20) | 55(19) | 96(16) | 36(22) | 66(23) | 139(23) | 19(12) | 42(14) | 100(16) | 10(6) | 7(2) | 58(10) |
| **LATE SETTING** | 55(41) | 99(36) | 182(35) | 37(28) | 79(28) | 98(19) | 29(22) | 58(21) | 137(26) | 7(5) | 34(12) | 85(16) | 6(4) | 9(3) | 24(5) |
| **PHARMACOTHERAPY** | 106(51) | 207(46) | 396(42) | 50(24) | 123(27) | 181(19) | 23(11) | 64(14) | 199(21) | 17(8) | 47(10) | 123(13) | 13(6) | 12(3) | 44(5) |
| **RADIATION** | 22(35) | 51(36) | 78(30) | 14(22) | 33(24) | 45(17) | 12(19) | 29(21) | 65(25) | 11(17) | 23(16) | 44(17) | 4(6) | 4(3) | 25(10) |
| **GENETIC ALTERATIONS** | 0 | 0 | 12(17) | 2(29) | 0 | 7 (10) | 2(29) | 3(60) | 27(39) | 2(29) | 1(20) | 16(23) | 1(14) | 1(20) | 7(10) |
| **SURGERY** | 14(18) | 23(18) | 31(13) | 14(18) | 28(21) | 29(13) | 29(38) | 49(38) | 55(24) | 15(20) | 29(22) | 78(34) | 4(5) | 1(1) | 38(16) |
| **PATHOLOGY** | 37(42) | 42(30) | 99(32) | 19(22) | 19(14) | 49(16) | 17(19) | 42(30) | 67(22) | 9(10) | 28(20) | 62(20) | 5(6) | 7(5) | 30(10) |
| **SCREENING** | 1(100) | 7(39) | 10(26) | 0 | 1(6) | 3(8) | 0 | 4(22) | 14(36) | 0 | 3(17) | 6(15) | 0 | 4(22) | 6(15) |
| **DIAGNOSTIC** | 2(14) | 8(11) | 31(16) | 3(21) | 13(18) | 23(12) | 4(29) | 31(43) | 51(26) | 4(29) | 17(24) | 62(32) | 1(7) | 3(4) | 28(14) |
| **DISEASE STAGING** | 2(11) | 10(15) | 53(22) | 3(17) | 6(9) | 28(12) | 11(61) | 38(58) | 71(30) | 2(11) | 7(11) | 66(28) | 0 | 5(8) | 20(8) |
| **FOLLOW UP** | 2(9) | 7(12) | 13(10) | 3(14) | 7(12) | 13(10) | 6(27) | 16(28) | 36(27) | 4(18) | 13(23) | 31(23) | 7(32) | 14(25) | 39(29) |
| **TRANSPLATATION** | 0 | 10(31) | 13(19) | 2(67) | 9(28) | 9(13) | 1(33) | 8(25) | 24(35) | 0 | 4(12) | 19(27) | 0 | 1(3) | 4(6) |

**Table S4**

**Types of recommendation and associated grade of recommendation**

| **TYPE OF RECOMMENDATION** | **A**  **First**  **n (%)** | **A**  **Previous**  **n (%)** | **A**  **Current**  **n (%)** | **B**  **First**  **n (%)** | **B**  **Previous**  **n (%)** | **B**  **Current**  **n (%)** | **C**  **First**  **n (%)** | **C**  **Previous**  **n (%)** | **C**  **Current**  **n (%)** | **D**  **First**  **n (%)** | **D**  **Previous**  **n (%)** | **D**  **Current**  **n (%)** | **E**  **First**  **n (%)** | **E**  **Previous**  **n (%)** | **E**  **Current**  **n (%)** |
| --- | --- | --- | --- | --- | --- | --- | --- | --- | --- | --- | --- | --- | --- | --- | --- |
| **RARE CANCER** | 28(31) | 88(31) | 250(37) | 50(56) | 127(44) | 273(40) | 11(12) | 55(19) | 117(17) | 0 | 16(6) | 25(4) | 0 | 0 | 9(1) |
| **SOLID CANCER** | 137(39) | 313(45) | 649(45) | 165(47) | 278(40) | 493(34) | 41(12) | 77(11) | 198(14) | 10(3) | 21(3) | 51(4) | 1(0,3) | 2(0,3) | 31(2) |
| **HAEMATOLOGY CANCER** | 2(33) | 62(32) | 142(36) | 4(67) | 94(48) | 179(45) | 0 | 27(14) | 56(14) | 0 | 11(6) | 16(4) | 0 | 0 | 3(1) |
| **SUPPORTIVE THERAPY** | 12(32) | 19(34) | 25(33) | 20(54) | 28(51) | 37(49) | 3(8) | 6(11) | 12(16) | 2(5) | 2(4) | 0 | 0 | 0 | 1(1) |
| **EARLY SETTING** | 72(45) | 145(50) | 292(48) | 63(39) | 108(37) | 188(31) | 19(12) | 26(9) | 84(14) | 6(4) | 8(3) | 23(4) | 1(1) | 2(1) | 20(3) |
| **LATE SETTING** | 52(39) | 107(38) | 204(39) | 69(51) | 132(47) | 226(43) | 12(9) | 30(11) | 78(15) | 1(1) | 9(3) | 15(3) | 0 | 0 | 2(0,4) |
| **PHARMACOTHERAPY** | 91(43) | 190(42) | 423(45) | 91(43) | 199(44) | 350(37) | 22(10) | 48(11) | 126(13) | 4(2) | 14(3) | 27(3) | 1(0,5) | 1(0,2) | 16(2) |
| **RADIATION** | 20(32) | 64(46) | 93(36) | 35(56) | 56(40) | 98(38) | 7(11) | 15(11) | 47(18) | 1(2) | 4(3) | 8(3) | 0 | 1(1) | 10(4) |
| **GENETIC ALTERATIONS** | 2(29) | 2(40) | 27(39) | 4(57) | 2(40) | 21(30) | 0 | 0 | 18(26) | 1(14) | 1(20) | 3(4) | 0 | 0 | 0 |
| **SURGERY** | 21(28) | 52(40) | 90(39) | 45(59) | 61(47) | 93(40) | 9(12) | 14(11) | 34(15) | 1(1) | 3(2) | 11(5) | 0 | 0 | 3(1) |
| **PATHOLOGY** | 42(48) | 66(48) | 154(50) | 37(42) | 47(34) | 105(34) | 8(9) | 21(15) | 34(11) | 0 | 4(3) | 8(3) | 0 | 0 | 5 (2) |
| **SCREENING** | 0 | 4(22) | 12(31) | 1(100) | 4(22) | 17(44) | 0 | 7(39) | 7(18) | 0 | 3(17) | 0 | 0 | 0 | 3(8) |
| **DIAGNOSTIC** | 5(36) | 40(56) | 98(50) | 6(43) | 66(34) | 60(35) | 3(21) | 24(12) | 22(13) | 0 | 0 | 5(3) | 0 | 0 | 2(1) |
| **DISEASE STAGING** | 6(33) | 30(45) | 115(48) | 10(55) | 27(41) | 68(29) | 1(6) | 7(11) | 37(15) | 1(5) | 2(3) | 12(5) | 0 | 0 | 4(2) |
| **FOLLOW UP** | 6(27) | 15(26) | 31(23) | 12(54) | 31(54) | 62(47) | 2(9) | 6(10) | 31(23) | 2(9) | 5(9) | 7(5) | 0 | 0 | 1(1) |
| **TRANSPLATATION** | 0 | 9(28) | 23(33) | 3(100) | 17(53) | 34(49) | 0 | 4(12) | 8(12) | 0 | 2(6) | 4(6) | 0 | 0 | 0 |
